# American Academy of Dental Science

**Published:** 1901-06

**Authors:** 

**Affiliations:** American Academy of Dental Science


### AMERICAN ACADEMY OF DENTAL SCIENCE.

THE regular monthly meeting of the American Academy of Dental Science was held at Young's Hotel, Boston, on Wednesday evening, March 6, 1901, at six o'clock, President V. C. Pond in the chair.

The minutes of the previous meeting were read and approved.

Previous to the dinner Dr. Meriam, in answer to several inquiries by Fellows of the Academy regarding the condition of the membrane where extension crowns had been inserted, presented a patient showing the case as reported before the Academy October 5, 1898, and published in the INTERNATIONAL DENTAL JOURNAL, March, 1899. The crowns had been in position over eight years, and the healthy condition of the membrane and of the teeth adjoining them, together with their cleanliness and the parts adjoining, could be seen. The case elicited much interest and favorable comment.

The post-prandial exercises were opened with an essay by G. N. P. Mead, M.D., of Winchester, Mass., entitled "A Case of Swallowing a Tooth."

(For Dr. Mead's paper, see page 380.)

### DISCUSSION.

*Dr. Mead.*—I had an idea that what I have presented to you was something unusual, but I find, in talking with some of your members, although it has a very small space in literature, it may not be so very rare a thing.

*Dr. Briggs.*—I would like to ask Dr. Mead if he thinks the X-ray would have shown that tooth, and if so, what he would have done if he had discovered it there?

*Dr. Mead.*—I should think the X-ray would have revealed it in the beginning. Very likely after the pneumonia it might have been difficult to see, on account of thickening, which must have remained as long as the tooth was in the bronchus. The X-ray could be used antero-posteriorly through the chest wall, but I doubt if it could give the right angle of revelation which would be necessary to locate it for any surgical procedure. As to what could be done early, opening the trachea, with irritation of the bronchus, thereby hoping to have the tooth coughed out, seems to be the best method.

*Dr. Williams.*—I think that a very remarkable case, and it has been very clearly described, so that there will be no difficulty in anybody's understanding the case thoroughly. I am very glad we are to have it published in our transactions, in our journal. It will certainly be a rather notable thing to have on record.

*Dr. Mead.*—Mr. President, may I add one case that was of interest to me? It was a suit brought against a dentist who broke the crown of a tooth in the forceps and it was drawn into the trachea, the person being under the influence of gas. The piece of tooth remained in the lung one week, during which time the man had paroxysms of coughing and expectoration of blood. It was finally coughed up. He sued the dentist and recovered five hundred dollars. The decision was appealed, but the higher court sustained the verdict.

*Dr. Werner.*—Had the patient any suspicions of having swallowed the tooth?

*Dr. Mead.*—No, sir.

*Dr. Werner.*—And no medical diagnosis suggested anything of the complaint?

*Dr. Mead.*—No, sir. The pneumonia occurred in two days, and it was supposed to be from sucking blood into the lung. Both the dentist and the physician thought it was due to this.

*Dr. Werner.*—Infection?

*Dr. Mead.*—Yes.

*Dr. Williams.*—What was the anæsthetic?

*Dr. Mead.*—Nitrous oxide gas.

*President Pond.*—The subject is passed.

### MAKING GOLD INLAYS BY MODELS.

*President Pond.*—Dr. Andrews will show Dr. George S. Allan's method of making gold inlays by models.

*Dr. Andrews.*—Mr. President and gentlemen, I think it was some time during last December that Dr. Allan sent me some of his gold boxings, which I was to show at the January meeting, but from a crowded condition of the evening's programme it was found that we had no time. It was advertised by card to be shown at the February meeting, but the same condition prevailed, and it was carried over to this meeting. In the mean time the whole thing has been published, and probably most of you have read the account of how Dr. Allen makes his gold boxings.

For fear that some may not have seen this, I will simply say that a cavity presents itself, and is shaped so that an impression taken will give a clear impression of the cavity. If there are any undercuts they are filled up with cement, or with impression material, so that the impression will come out clear. All surfaces are carefully prepared so as to be at right angles, with good substantial edges. The impression is then taken to the laboratory, a plaster model is made, and from that a cast with Melotte's metal, and a base-piece, fitting the bottom of the cavity, is struck out of No. 30 gold,—pure gold I think he uses. This takes but a little while. After it is struck up he uses a little solder, flowed on the bottom, to make it stiffer. It is then taken to the tooth and the fit is verified. Then modelling composition is placed upon it and it is shaped to the contour of the tooth. Enough is taken off to allow for the thickness of the No. 30 gauge gold cover, which I think is made of 20-carat gold, to go between the bite and the composition which is used to contour the boxing. This is taken to the laboratory, another die is made and the contour piece struck up, the impression materials removed, and the two are then soldered together and filed down to make the boxing, as he calls it, so that it will exactly fit the cavity.

Dr. Allan has sent for our inspection some of these boxings which he has made, and they are very beautiful. They are as fine as the finest gold filling to look at. And I have the models with me, which I shall now pass around.

I will say that I do not think it is always necessary to get up dies, for I have, in a plaster cast, with cotton or spunk, driven some soft pure gold to the bottom of the cavity, thus forming the base-piece, then put on my modelling composition, shaping the cavity as I thought it ought to be shaped, and have then burnished the gold on this without any dies at all, thus making the outer piece. I have made and put in two or three boxings which have given very good satisfaction.

Dr. Allan had tried a number of the porcelain inlays on the back teeth, and found that they chipped and broke so badly in many cases that he devised this method of gold inlays, or boxings. I do not know whether it is wholly his own or not.

The pieces which I shall first show I have arranged in a box, numbered one, two, three, and four, showing first the small impression cup, which is made of thin steel which we band teeth with; it is made somewhat larger than the cavity. A little warm impression material is pressed into the cavity, allowed to cool, and removed, and there you have your impression. The next piece is the impression in the impression cup. The third piece is the plaster cast made from the impression, with the base-piece stamped up in it. The fourth piece is a piece with modelling composition arranged to form the contour, and the fifth piece is the outside piece struck up to fit over that.

*Dr. Williams.*—How is it retained in the cap?

*Dr. Andrews.*—I am glad you asked the question. When these two pieces are soldered together, of course it makes a little box on the hollow capping or inlay. Dr. Allan then files openings on the inside surface sufficiently large to let cement run in, and his idea is to fill the boxing with cement, placing it within the boxing and pressing it home with the finger, holding it while it hardens. He has had very good success. I have three teeth with the boxings here, which Dr. Allan made, and I have had my assistant put a thread around them so that you can take them out and look at them, and not run the risk of losing them. Kindly pass them around, and you can see how nicely they are made and how well they fit.

*Dr. Bradley.*—Are these Dr. Allan's?

*Dr. Andrews.*—These are Dr. Allan's, sent to me to show to the Academy nearly three months ago.

*Dr. Bradley.*—After fitting the impression with modelling composition you put on a pure piece of gold, and then you have to take it off to get the modelling composition out?

*Dr. Andrews.*—Yes.

*Dr. Bradley.*—How do you put it on again after you take it off?

*Dr. Andrews.*—It is fitted down so perfectly that it will fit in one place only.

*Dr. Bradley.*—What solder do you use?

*Dr. Andrews.*—Two or three grades lower than the gold.

*President Pond.*—Is there anything to be said on this subject of inlays? Quite a number of you have seen them.

*Dr. Ames.*—I have something here in that line which I will pass along. This is not in competition at all. I prepared this some four years ago to bring up, but I do not think it is as good as the one that you see, made by Dr. Allan. It is simply a piece of 28-gauge pure gold, contoured over a cavity and a bar soldered across, and then the whole is cemented in. You will see one tooth that has the inlay before it is set with cement, and the other is where it is fastened to the tooth with cement. I formed it around, covering the thin edge of the tooth, grinding the edge up, contoured it with contouring pliers, and then cemented the edge of the cap; after the cement is hardened so that it is firm, I burnished the edge down so that it is closed. I have some that have been in use from two to eight years. Some have done very well, and a few have failed.

*Dr. Andrews.*—Mr. President, I will say that Dr. Allan has used these cappings, but he found in cases of hard mastication that the edges would come away and turn up, and it was that very thing that set him to thinking how he could make something with edges absolutely solid, so that there would be no breaking away or turning up from hard mastication. He says he thinks that this boxing meets the case perfectly.

*Dr. Ames.*—I will freely say that I think Dr. Allan's is a good deal better.

*Dr. H. A. Baker.*—Since Dr. Andrews described this method of Dr. Allan's, it reminds me of a method which I thought was quite unique. About a year ago I went before the Vermont State Dental Society. While there Dr. R. M. Chase, of Bethel, presented a method which seemed to me very practical in a great many cases, especially where a patient is unable to undergo the tedious operations of large gold fillings.

After preparing his cavity, without the undercuts, an impression was taken with a moulding preparation very similar to Melotte's mouldine. This gave him an impression of the cavity and a portion of the tooth surrounding it. In this impression was cast some fusible alloy, which gave him a fac-simile of the cavity and a sufficient amount of the tooth to get a good periphery and contour. This metal mould he takes to the laboratory and finishes as if it were in the tooth. He then removes it, and has a filling that exactly fits the cavity in the mouth. Then, before inserting it, he makes his undercuts in the cavity, also in the filling. It is then cemented in as an inlay, the same as Dr. Allan's is.

I would also say, Dr. Allan's specimens are very beautifully done, and I think practical, but it seems to me more complicated than Dr. Chase's, where one accomplishes the same results.

*Dr. Andrews.*—Mr. President, I think Dr. Baker would be surprised to see how simple it is. It requires nice judgment and nice work.

I would say, in regard to the solid gold inlay, I think Dr. Russell, of Keene, N. H., has used them a number of years. He was one of the first. I have seen several that Dr. Russell made four or five years ago,—the solid gold inlays.

*Dr. Baker.*—I have seen his inlays. This method seemed a very unique one to me.

*Dr. Meriam.*—Mr. President, I think the forerunner of all this work, like almost every good thing, and many bad ones, started in Salem. While I am not going to say much about it, it was done by Dr. Fiske. I think he began making caps perhaps thirty years ago, and his practice at that time was to melt a globule of pure gold, place it on a little appliance he had, and flatten it out. In it was a small hole that a portion of the gold entered. The appliance was then reversed, he drove a punch in that spread it, and made it virtually in the form of a split rivet. Then he pared the edges and fitted the cavity. He also used pins, put on like Dr. Ames has used them, set in gutta-percha. But you know, if there is movement, gutta-percha may loosen in its attachment, and there was a tendency for it not to adhere, and later the cap curled up at the edges. But I occasionally see them now that were done at that time. I think Dr. Williams knows more about that than I, and I wish he would tell us.

*Dr. Williams.*—I think it was shortly after the society was formed that Dr. Fiske, of Salem, was a member, and a very skilful man he was in some lines. He introduced an appliance for striking up these inlays. The metal of which the studs were made was soft gold. I have one of his machines now. It must be pretty near the date that Dr. Meriam mentions. It was a very simple matter to strike up the studs. The trouble was in the anchorage: that was not sufficiently stiff, a little yielding, so that in some cases in mastication it would give way, and thus it rather fell into disuse.

But still there were advantages. In a number of cases I know those fillings or cappings lasted a long time, with great benefit to the teeth, where, indeed, I think, especially in some large cavities, a solid gold filling might have injured the safety of the tooth from its hard pressure against the walls of the tooth, and, as is often the case, from its frequent expansion by contact with hot substances. The expansion of gold under the influence of heat often breaks down a tooth, and we do not understand why it is. This of course is not subject to that danger, and I think in many cases it preserved the tooth longer than a solid gold filling would.

If I had known this subject was to be brought up, I would have brought this appliance which I have. It is a simple little thing, but very effective.

*Dr. Meriam.*—Since I sat down I remember that I read a paper before the Academy on this subject, because I remember the capping being claimed by some one in New York. And Dr. Williams was kind enough at that time to bring the little appliance that Dr. Fiske had made and show it in the Academy. I know it came in a chamois-skin pouch.

### A NEW TOOTH-BRUSH.

*President Pond.*—Anything further? If not, we will pass to the next subject. Some new appliances will be shown by Dr. H. F. Hamilton.

(For Dr. Hamilton's paper, see page 377.)

*Dr. Hamilton.*—Are there any questions you would like to ask about that?

*Dr. Werner.*—Do I understand you to claim that all brushing of the gum is injurious, whether done rightly or wrongly?

*Dr. Hamilton.*—I do not claim that *right* brushing is *wrong*.

*Dr. Werner.*—But *wrong* brushing is not *right*. That is exactly what I wanted to say.

*Dr. Hamilton.*—I merely claim that I have seen a great deal of harm done by brushing the teeth wrongly.

*Dr. Briggs.*—I think Dr. Hamilton could almost answer that question that way. It seems to me from my observation that I should agree with him entirely, that I never had seen a case of recession of the gum, pure and simple (I do not mean the pockets that we have from pyorrhœa, where later the gum becomes destroyed), that was not invariably due to the misuse of the tooth-brush or the floss-silk. A great many of the ills that teeth are heir to to-day are due to the abuse of those things. I have no doubt that Dr. Hamilton's brush is to be a great help in such cases.

Of course, the thing is to see that patients use the brush properly when they do use it, and not use it as a scrubbing-brush. As I tell them, the brush is simply a vehicle for carrying powder or antiseptic liquid to all parts of the mouth, and not particularly to the teeth. And when people wish to shine their teeth, they should do as they do when they wish to shine their nails, use a buffer, and not a brush. You could not get a polish on your nails with a brush, and you do not get the best polish on your teeth with a brush.

*Dr. Williams.*—The brush seems to be very well devised for the purpose, if the badger's hair will not mat down between the rows of bristles. That of course can be told from experience. Does the badger's hair mat down?

*Dr. Hamilton.*—It does not mat down. It wears off rather rapidly.

*Dr. Williams.*—In that case the question would be whether it would become more like a piece of felt than an elastic brush made of small bristles, but elastic bristles, not so fine as badger's hair.

The best brush I have found has been Deponte's No. 1741, which I get by the quantity at Week & Potter's. They have four rows of bristles. The bristles are fine but elastic, so that they will retain sufficient stiffness to penetrate the crevices without matting down.

AN IMPROVED CROWN.

*Dr. Hamilton.*—I have a crown here that I showed some of the members some time ago. I have been using it since with considerable success. I have written down what I have to say about it.

(For Dr. Hamilton's description, see page 379.)

*Dr. Andrews.*—I would like to ask Dr. Hamilton how the trephine cuts the place in the tooth if it is an unequal surface; if it is rounded up, it must cut deeper on the sides.

*Dr. Hamilton.*—Yes, it cuts deeper on the side. The platinum band which goes in the root is not of equal length all the way around. In some cases it did not go all the way round, and on the labial surface there was no band at all, but that did not interfere with the general principle.

*Dr. Williams.*—I was going to suggest to Dr. Hamilton that his very ingenious application of the rubber tube suction struck me as capable of being employed on a still larger scale, perhaps in cases where it is desired to draw diphtheritic germs from the throats of children, with larger tubes; and I think the thing is greater than it seems from his presentation of it.

*Dr. Meriam.*—I wish to say also that that was one of the things Dr. Fiske did with his saliva-pump: he had some soft rubber disks that he put onto the end of the tube. But those things do not last forever.

*Dr. Bradley.*—I think the crowns have such merit that if we could have them illustrated in the INTERNATIONAL DENTAL JOURNAL it would be very valuable. The cuts should be made to show the different stages of the work. It would be of very great interest, I think, to the profession in general if they could be published.

*President Pond.*—Is there anything further to be said on any of these subjects?

MOUTH-MIRROR.

*Dr. Taft.*—Among all the instruments we use in every-day practice, there is nothing which gives me greater satisfaction than a first-class mouth-mirror. Such a mirror I presented at one of our meetings some time ago, and for the benefit of those who were not present I will pass this one around. It is made and sold by Dr. W. M. Sharp, of Binghamton, N. Y. The parts are easily adjustable, as you will notice, but the clearness of the lens is what I would particularly call your attention to. The price of the mirror, complete, is one dollar. Additional lenses, plain or magnifying, can be obtained at any time at one-third the price of the usually unsatisfactory mirror sold by the dental dealers.

I think this meeting is proving to be one of the most interesting that we have had for some time. It has to do with practical everyday subjects, and we are getting a good deal of useful information. I want to speak incidentally of an interesting case, to me at least, upon which I operated recently, for the reason that I was curious to observe what the result of the operation would be. The patient was a common, but vicious, red fox, and might with propriety be added to the collection of wild animals whose acquaintance we have made through the writings of that fascinating author, Mr. Ernest Seton-Thompson.

AN OPERATION ON A FOX.

A friend of mine, who is a breeder of foxes, consulted me recently with regard to a vicious male, who every other night or so would kill one of his companions in the enclosure upon the farm where they were being raised. Extraction or excision of the four tushes, upper and lower, would, I thought, be the means of curbing his murderous propensities. The latter was the course finally decided upon. The operation was not completed without plenty of exciting details, but, briefly told, the fox was first caught, then bagged and chloroformed. A pair of excising forceps quickly brought the four tushes to the level of the other incisors, and the pulps were then removed with a fine nerve-broach. A common rat-tail file served to nicely smooth and bevel the edges of the stumps, and to complete the operation. The next day found the animal suffering apparently no pain or other inconvenience. On the second night after the operation, however, he killed two more of his companions. About ten days after that he was seen to act queerly, running about in a circle and butting his head against stones and walls of the enclosure, and was himself found dead the next morning. While I regret to report the death of my patient, I am pleased to state, nevertheless, that, viewed from the usual surgical stand-point, "the *operation* was a success."

DISCUSSION.

*Dr. Andrews.*—I want to endorse what the doctor says about the Sharp mirror. I have one of those Sharp mirrors, and I have a number of other mirrors, but I always want that mirror. The glass is made by a microscope lens maker, and it is perfect. I never have had so much pleasure in using a mouth-mirror as I have in using those of Dr. Sharp's.

*Dr. Meriam.*—The subject of mirrors is up to-night, and it reminds me of what I put in my pocket. I have an ordinary magnifying mirror. I fancy it was made in Germany. I have seen it under various marks. I buy a good magnifying mirror with a white handle, etc., for between five and six dollars a dozen, and I can have a fresh one whenever I wish at a less cost than to have them repaired. I have accumulated a great stock of handles as a "by-product," which have been around the office, and I have been trying to utilize some of them within the past few weeks.

*Dr. Williams.*—Where do you get your mirrors?

*Dr. Meriam.*—I sound the different drummers that come along, and when one talks reasonably I buy of him. I used to get a very good mirror from Goldthwaite, the surgical instrument man, but I have not been in Boston so much of late years, and I have taken to buying them from people who come along.

You will see some of the handles are fitted to an ordinary chuck; others are broach-holders, explorers, etc.

### NEW RETAINING APPLIANCE.

*Dr. H. A. Baker.*—Mr. President, under the head of presentation of specimens I wish to present a new retaining appliance. Something like five years ago I brought before this society a new retaining device, calling it a new hygienic retaining appliance for both upper and lower teeth to hold them in place after regulating, the lower one consisting of a rubber plate fitting the gum from the sixth-year molars forward, the upper edge of it extending around from molar to molar about one-sixteenth of an inch below the gum line inside, two gold bands being fitted to each first bicuspid; upon this band a globule of gold solder flowed on the lingual side of the bands; then in this solder was filed a notch. In the rubber plate was anchored a platinized gold wire about the size of a common pin, running from the plate to the notches in the band with flattened ends; then the same kind of wire running from the plate up on and between the cutting edges of the cuspids and laterals, extending round, front and back of the four front teeth, also wires anchored into the plate extending upward to about one-half the length of the crowns of all the remaining teeth and bent at right angles, and the end resting against the side, to hold them into position. To prevent the back part of the plate from slipping down a wire was brought up and hooked over in the sulci of the sixth-year molars.

The appliance I present to-night is an improvement upon the above, and it differs in the following way (see Fig. 4, page 378): In place of the rubber plate is a half-round platinized gold wire about one-sixteenth of an inch wide, with the flattened side next to the gum, and the other wires are soldered to this in place of being anchored into the rubber. To firmly hold the appliance in position a gold band is fitted around each twelfth-year molar; on the lingual surface of each band is soldered a tube which stands in line with the teeth or perpendicular, and from the half-round gold wire is soldered another wire about one-thirty-second of an inch in size, hooking over and entering the tube at the top; then around each bicuspid is fitted gold bands; on the lingual surface of each band is soldered a tube in a horizontal position, or crosswise of the tooth; then this tube, about three-sixteenths of an inch in length, is split lengthwise. From the half-round gold wire on each side is another wire running up to the tube, and then a short piece of wire soldered to the end of it, forming a T, going into the slot and fitting accurately inside of the tube; then the four bands are cemented to the teeth, with the wires fitting around the front teeth as above stated. This can be adapted to the upper as well as to the lower jaw. It is not intended to be removable by the patient, but can be taken out by the dentist. It is my belief that this appliance can be worn a sufficient length of time to retain the teeth without injury, as there are no places for the secretions to lodge about the teeth, for it is perfectly self-cleansing.

*Dr. Meriam.*—Mr. President, I would like to speak of a matter that relates to the presentation of specimens, and that is, whether we cannot have a little more made of it. We have seen to-night how we are all interested, and how valuable it is. One of the rules of the Odontological Society of Great Britain is that gentlemen who are to present specimens shall send a notice to the proper officer a few weeks before the meeting, so that all the members may know that this gentleman is to present a specimen at that meeting, and it serves as a table of record of the specimens, and it also leads to persons who are interested coming to that meeting. I think if it were a possible thing for the Academy to adopt that rule, so that we may have that record of them on the card of the meeting, it would be a good thing. Of course, we cannot do that when we have long meetings with long papers, but we can do it in a meeting like this.

*Dr. Taft.*—Dr. Meriam's remarks only serve to emphasize what I intended to speak of. We have, as we all know, from time to time more or less of a surplus of money in our treasury, and I know of no better way of using that surplus, and thereby helping to improve the INTERNATIONAL DENTAL JOURNAL and to increase its circulation, than by appropriating, whenever it seems advisable, a sum of money sufficient to illustrate such specimens, for example, as have been presented here to-night. This would give added interest to the reports of our meetings as they appear in the INTERNATIONAL from month to month, besides making them often more intelligible to the general reader. If the Academy approves of this idea I shall be glad to offer a motion to that effect.

*Dr. Williams.*—I am in harmony with Dr. Taft's idea, and I have often thought that the INTERNATIONAL DENTAL JOURNAL would be more interesting if there were a greater abundance of illustrations of specimens, appliances, etc. I should quite concur in any plan by which surplus funds could be used for such illustrations, or whatever is judged necessary by the editor. If he makes that as a motion, I second it.

*Dr. Taft.*—I speak of this particularly, Mr. President, because I think we have not always been so judicious as we might be in our appropriations of money, and to express the hope that we may avoid similar mistakes in the future.

*Dr. Bradley.*—I said a few moments ago that if the crown Dr. Hamilton presented here this evening could be properly illustrated, it would add very much to the interest of the publication of the article. And I suppose if this retaining band, which is in two or three pieces (Dr. Baker: It is all one piece, except the band),—if the various parts of it could be illustrated, and then have it in position, it would make it very plain so that we could understand it. It would add a great deal to the description which we get from the stenographer's report. I should be very much in favor of judicious illustration.

*Dr. Meriam.*—I remember some years ago I was working up a directory, in connection with the Massachusetts Society, and I worked with the purpose of finding out what there was in Boston. My purpose was to list in the directory the name of every man in the section who would be of service to dentistry. We have fine machinists all over the city. We have a maker of fine tools up in Springfield. We have these things all around here. I say there is no spot in America where the resources of dentistry are better than right here in Boston. They have not got what we have anywhere else.

*Dr. Bradley.*—In order to bring this before the Academy, I will move you, sir, that the devices offered by Drs. Hamilton and Baker be illustrated, at an expense that will be satisfactory to the Executive Committee.

So voted.

On motion, adjourned.

Charles H. Taft,
*Editor American Academy of Dental Science.*

